# The Patient-Perceived Helpfulness of Measures Scale: Development and Validation of a Scale to Assess the Helpfulness of Using Measures in Psychological Treatment

**DOI:** 10.1177/10731911231195837

**Published:** 2023-09-28

**Authors:** Gina Di Malta, Mick Cooper, Julian Bond, Brett Raymond-Barker, Marsha Oza, Regina Pauli

**Affiliations:** 1 The Open University, Milton Keynes, UK; 2 University of Roehampton, London, UK

**Keywords:** scale development, routine outcome monitoring, clinical feedback, validity, Patient-Perceived Helpfulness of Measures Scale

## Abstract

In response to the increase in Routine Outcome Monitoring and Clinical Feedback, the Patient-Perceived Helpfulness of Measures Scale (ppHMS) was developed to assess the helpfulness—as perceived by patients—of using measures in psychological treatment. Study 1: The construct of *patient-perceived helpfulness of measures* was explored using thematic analysis with 15 patients. Six helpful and three unhelpful themes were identified and informed item development. Study 2: 28 items were formulated and rated by experts. Ten items were taken forward for psychometric shortening in a sample of 76 patients. Confirmatory factor analysis (CFA) led to an adequately fitting six-item model with excellent internal consistency, and convergence with the Delighted-Terrible single item of product satisfaction and a single item of measure helpfulness. Study 3: In a stratified online sample of 514 U.K. psychotherapy patients, a five-item model constituted the best fit. The final ppHMS had excellent internal consistency (McDonald’s ω = .90), convergent validity with psychotherapy satisfaction (*r* = .5; *p* < .001), divergence from social desirability (*r* = .1), and metric and scalar invariance across measures. Study 4: Analyses were replicated and confirmed in a stratified U.S. sample (*n* = 602). The ppHMS is a reliable and valid scale that can be used to assess and compare patients’ perceptions of the helpfulness of different measures as part of their psychological treatment.

## Clinical or Methodological Significance of This Article

The Patient-Perceived Helpfulness of Measures Scale (ppHMS) is a self-report measure developed and validated to support practice and research on routine outcome monitoring (ROM) and clinical feedback (CF) across mental health services. The ppHMS can be used to identify the measures that patients perceive as most helpful and unhelpful. This can support decisions on what measures should be used in ROM and CF systems, as well as tailoring these systems to the unique preferences of each patient.

## Introduction

There has been a rapid development in the implementation of Routine Outcome Monitoring (ROM) and Clinical Feedback (CF) in psychotherapy and mental health practice over the past two decades internationally (e.g., Lambert et al., 2018; Muir et al., 2019; Roe et al., 2015). ROM and CF have become recommended clinical practice (Lambert, 2017). ROM and CF typically rely on standardized outcome and process measures, which are usually administered regularly throughout therapy (e.g., Lambert & Harmon, 2018). Lambert and Harmon (2018) propose that ROM practice should include a measure of patient functioning that has established validity, reliability, and appropriate norms, with a practical mode of administration, that can be scored clearly, and is brief (i.e., require no more than 5–10 minutes of time). Several systems are currently in use, each assessing different aspects of treatment outcome (Kendrick et al., 2016). For instance, the Partners for Change Outcome Management System (PCOMS) consists of the Outcome Rating Scale (ORS), which assesses well-being in daily life on an interpersonal and intrapersonal level as well as on social roles, and the Session Rating Scale (SRS), which assesses the therapeutic alliance aspects as defined by Bordin (1979; Duncan et al., 2003; Miller et al., 2003; Sparks & Duncan, 2018). In the UK National Health Service’s Improving Access to Psychological Therapies program, the most commonly used measures for ROM include the Generalized Anxiety Disorder (GAD-7; Spitzer et al., 2006), Patient Health Questionnaire (PHQ-9; Kroenke & Spitzer, 2002), and the Clinical Outcomes in Routine Evaluation Outcome Measure (CORE-OM; Barkham et al., 2013).

Evidence suggests that the use of ROM and CF in clinical practice has a significant impact on mental health outcomes (e.g., Bovendeerd et al., 2022; Brattland et al., 2018; de Jong et al., 2021; Lambert et al., 2018). For instance, in a randomized controlled trial (RCT) of 170 adult patients, ROM was associated with better treatment outcomes independent of patients’ initial levels of psychological distress (Brattland et al., 2018). ROM and CF are hypothesized to contribute to treatment outcomes primarily by providing clinicians with information that can help redirect treatment and prevent negative outcomes (e.g., Lambert, 2010). It is also possible that patients’ monitoring of treatment progress, itself, may contribute to improved outcomes. While evidence from the Outcome Questionnaire (OQ) is equivocal on this (see Lambert & Shimokawa, 2011), research using goal attainment measures has indicated that some patients found regular monitoring guided and reinforced feelings of progress (Di Malta et al., 2019). Such positive impacts of goal monitoring are also evidenced in the wider population (Harkin et al., 2016).

However, research also indicates that ROM is not beneficial to all patients (de Jong et al., 2018; Errázuriz & Zilcha-Mano, 2018). In addition, effects for certain groups of patients, such as children and young people, remain unclear (Bergman et al., 2018). Thus, there remains a critical question of how ROM and CF systems might be improved.

Understanding and assessing patients’ perceptions of the helpfulness of ROM and CF measures may be one means of developing the effectiveness of these systems. This is both at the individual patient level and at the more macro, service, or population level. With respect to the former, assessment of *patient-perceived helpfulness of measures* during psychotherapy—with the potential to adjust the type or number of measures being used, as with the Norse Feedback CF system (Hovland et al., 2020)—can be considered a way of assessing (and, subsequently, accommodating) patients’ activity preferences: a practice associated with reduced dropout and improved treatment outcomes (Lindhiem et al., 2014; Swift et al., 2018; Windle et al., 2020). Norcross and Cooper (2021) suggest that assessing and accommodating such preferences may be beneficial for three reasons: (a) *matching effects*, whereby patients have some awareness of what is most helpful and unhelpful to them; (b) *choice effects*, whereby the stating of preferences leads to feelings of empowerment; and (c) *alliance effects*, whereby preference assessment and accommodation leads to enhanced feelings of trust, safety, and feeling listened to in the psychotherapy relationship (Cooper et al., 2023; Handelzalts & Keinan, 2010). Assessing patient-perceived helpfulness of measures can be considered part of a wider movement in the health care field toward “personalized medicine” (Norcross & Cooper, 2021)—now established as a cornerstone of evidence-based practice (American Psychological Association [APA], 2006).

With respect to enhancing outcomes at more macro levels, assessing patients’ perceptions of form helpfulness can help services, commissioners, policy-makers, and researchers to adopt and develop ROM and CF systems that are perceived as most helpful by patients—or by particular groups of patients (e.g., patients with particular diagnoses, or in particular marginalized communities). Not only can this contribute to outcomes through matching mechanisms, but it can be considered an ethical responsibility for psychotherapy providers: involving service users in the design and development of their own mental health provisions (Thornicroft & Tansella, 2005).

Evidence on patients’ experiences regarding the helpfulness and unhelpfulness of ROM and CF can support an understanding of patient-perceived helpfulness of measures (e.g., Solstad et al., 2019). One common issue known to arise is *response burden* or *participant fatigue—*the effort required by the patient to answer a form (Speight & Barendse, 2010). Known factors that affect patients’ response burden include the cognitive load required, and the layout and interface of the reporting format (Eisele et al., 2022). A recent meta-analysis suggested a significant association between length of form and dropout (Rolstad et al., 2011). In addition, ROM tended to be experienced as unhelpful by triggering suspicion toward service providers, which could be detrimental to the therapeutic process (Solstad et al., 2019). With regard to more specific experiences of patient-perceived helpfulness of measures, a systematic literature search identified 16 qualitative studies of patient experiences of ROM in mental health services. Here, helpful aspects were flexibility around form use and the capacity of measures to capture the complexity of the patient’s issues (Solstad et al., 2019). ROM was also perceived as most helpful when they empowered patients and were accompanied by collaborative practice (Solstad et al., 2019). Similarly, in a video-assisted interpersonal process recall study with 12 psychotherapy patients, helpful experiences of ROM were associated with flexibility (e.g., measures reflecting patients’ needs or preferences) and with patients knowing that ROM would be used in a meaningful way (e.g., how clinicians would make use of it) (Solstad et al., 2021). In adults with psychosis, helpful experiences of ROM included feeling understood, being provided with opportunities to reflect and express feelings, and tracking progress toward goals (Fornells-Ambrojo et al., 2017).

Regarding the helpfulness of specific measures, most users reported that they benefited from using the CORE-10 before and after therapy, and valued the opportunity to keep track of their progress (Rodgers, 2018). However, some users suggested that they found the CORE-10 restrictive, simplistic, impersonal, or irrelevant (Rodgers, 2018). Two studies have compared the helpfulness to patients of using various measures as part of their treatment using a single-survey item of form helpfulness (e.g., Cooper et al., 2015; Fornells-Ambrojo et al., 2017). In Cooper et al.’s (2015) study, for instance, the perceived helpfulness of the Goals Form, a goal-based outcome measure, was 4.2 on a 5-point scale. By contrast, the mean rating of the PHQ-9 was 3.7.

## The Program of Research

To date, there is limited evidence on what constitutes patient-perceived helpfulness of measures; there is no concise, easy-to-use, reliable, and valid form of measurement that can be used across (a) the individual patient level, to assess patients’ experiences of completing measures and, potentially, adjust the ROM and CF systems being used accordingly and (b) at the service- or population-level—for research, evaluation, service development, and commissioning—to identify patients’ overall perceptions of form helpfulness, as well as the relative helpfulness of different measures. Single-survey items have provided an approximate indication of patient-perceived helpfulness of measures (e.g., Cooper et al., 2015), but lack the necessary characteristics to be established as internally reliable measures of this construct (DeVellis & Thorpe, 2021). The current project is a collection of four interlinked studies aimed at developing and validating a brief measure, the Patient-Perceived Helpfulness of Measures Scale (ppHMS), to assess the helpfulness to patients of using psychotherapy measures.

In this program of research, we aimed to answer the following research questions: (a) How do patients conceptualize psychotherapy form helpfulness? (Study 1). (b) Is a measure developed to assess patient-perceived helpfulness of measures, the ppHMS, reliable: Is internal consistency acceptable? (Cronbach’s alpha > .7) (Studies 2, 3, and 4); (c) Can we find a stable unidimensional factor structure for the ppHMS? (Acceptable fit statistics with CFA; chi-square test of exact fit, root mean square error of approximation [RMSEA ≤ .08], Tucker–Lewis index [TLI ≥ .95], comparative fit index [CFI ≥ .95], standardized root mean square residual [SRMR ≤ .08], and weighted root mean square residual [WRMR ≤ 1.0]) (Studies 2, 3, and 4). (d) Is the ppHMS a valid measure of patient-perceived helpfulness of measures: Is it divergent (construct validity) with a measure of social desirability, the Brief Social Desirability Scale (*r* < .1)? (Studies 3 and 4). Is it convergent with a single item of form helpfulness (*r* > .5) (Studies 2, 3, and 4). Is it convergent with a measure of product satisfaction, the Delighted-Terrible scale (*r* > .5) (Studies 2, 3, and 4). Is it convergent with a measure of service satisfaction, Satisfaction With Therapy and Therapist–Revised (STTS-R; *r* > .3) (Studies 3 and 4). Is the latent variable invariant across different measures (Studies 3 and 4) and is it also invariant across time (Study 4)?

The research project was submitted for ethics consideration under the reference PSYC 15/164 in the Department of Psychology and was approved under the procedures of the University of Roehampton's Ethics Committee. We report how we determined our sample size, all data exclusions, all manipulations, and all measures in the study.

## Study 1: Qualitative Inquiry Into *Patient-Perceived Helpfulness of Measures*

In scale development, it is essential to adequately define the construct domain before developing items for a scale (MacKenzie et al., 2011). Study 1 is a qualitative inquiry into the construct of *patient-perceived helpfulness of measures* using interviews with psychotherapy patients. These inductive methods can support theory development where there is a lack of research on a construct (DeVellis & Thorpe, 2021).

### Procedure

Interviews were conducted at a free university psychotherapy research clinic that offered up to 24 sessions of integrative therapy for depression. Inclusion and exclusion criteria were set by the clinical service in which the study was conducted and were being 18 years old or over and having a PHQ-9 score consistent with a diagnosis of depression (PHQ-9 ≥10, Kroenke et al., 2001). Exclusion criteria were severe mental health conditions, including individuals experiencing psychosis, severe personality disorders, or drug and alcohol addictions.

In this setting, the PHQ-9 and GAD-7 were used at every session to monitor symptoms and outcomes. These measures are part of the UK Department of Health’s National Minimum Data Set (2011). Their use is supported by the National Institute for Health and Care Excellence for assessing clinical progress in mental health services (NICE, 2011). Psychotherapists had received training on introducing these measures to patients.

Semi-structured interviews were conducted by the first and second author after therapy had ended. Interviews lasted 45 minutes and explored the construct of patient-perceived helpfulness of measures by asking psychotherapy patients about the helpfulness and unhelpfulness of using two measures, the PHQ-9 and GAD-7, on a weekly basis as part of their treatment. Patients were asked about helpful and unhelpful aspects of using these measures in their psychotherapy. The researchers prompted patients’ memory by showing them the measures and discussed their content. Interviews were audio-recorded and transcribed.

### Material

#### Patient Health Questionnaire

PHQ-9 is a nine-item measure of depressive symptoms (Kroenke et al., 2001). Each item is rated using four ordinal response options (0, *not at all*; 3, *nearly every day*), giving a severity score between 0 and 27. PHQ-9 also rates difficulty in functioning. A score greater than 9 indicates clinically significant depression. The PHQ-9 is well validated against standard criteria, demonstrates sensitivity to change, and is used in a variety of clinical settings (Horton & Perry, 2016; McMillan et al., 2010).

#### Generalized Anxiety Disorder

GAD-7 is a seven-item measure of anxiety symptoms (Kroenke et al., 2007). Each item is rated on the same four ordinal responses as the PHQ-9, giving a severity score between 0 and 21. A score above 7 is recommended to identify a likely anxiety disorder.

### Participants

There were 15 participants, three males, 12 females. Ages ranged from 21 to 57, with a mean age of 31 years. Ten were from a White British ethnic group, one was Indian, one was African, one was mixed race, one identified as “other,” and one did not disclose their ethnicity. Participants had an average of 21 psychotherapy sessions. The majority (11 out of 15) had shown reliable improvement on the PHQ-9 at the end of their treatment course. There were five psychotherapists involved in the treatment of these 15 patients. Two were fully qualified counseling psychologists (had completed their doctoral training in Counseling Psychology and were accredited), two were counseling psychologists in doctoral training, and one was a counselor in training (MA level). One counseling psychologist saw two patients, one saw three patients, the two counseling psychologists in training saw three patients each, and the counselor in training saw four patients.

### Analysis

The analysis process comprised six phases as per Braun and Clarke’s (2006) thematic analysis. First, the interviews were transcribed by the fifth author and read several times by the first and fifth author. In a second phase, initial codes were generated by the fifth author, reflecting basic patterns of meaning identified in the text. These were audited by the first author and discrepancies were resolved in discussions. A third phase consisted of organizing the codes into broader themes and subthemes, under two overarching domains: (a) helpful aspects of using therapy measures and (b) unhelpful aspects of using therapy measures. At this point, themes and subthemes were named and defined. All the collated coded extracts in each theme and subtheme were re-read by the fifth and first author to consider whether they fitted the theme/subtheme and formed coherent units, followed by reporting.

### Results

Ten patients reported on the helpfulness of using the PHQ-9 and GAD-7 (Table 1). Eight patients said that the measures were overall helpful because they were “effective,” “good to do,” or patients had “liked it.” Six patients reported that filling out the measures supported self-reflection in psychotherapy. Five found them helpful to monitor their progress, for example, “good to track emotions.” Two said they were easy to use and easy to understand. Two reported that they thought the measures would be useful for their psychotherapist to help them.

**Table 1:** Helpful and Unhelpful Aspects of Using Therapy Measures (Study 1)

Domains	Helpful aspects of using therapy measures (*n* = 10)	Unhelpful aspects of using therapy measures (*n* = 8)
Themes and sub-themes	1. Helpful (*n* = 8) “effective,” “good to do,” “liked it,” “helpful”	1. Uncomfortable/upsetting (*n* = 3) “opens things up,” “difficult questions”
	2. Supports self-reflection (*n* = 6)	2. Already known and only helpful for the therapist (*n* = 3)
	3. Helpful to see progress (*n* = 5) “helpful to track progress,” “good to track emotions”	3. Inaccurate representation of experience (*n* = 2) “vague/unfit for purpose,” “unreliable,” “reflects what I want my score to be”
	4. Easy to use and understand (*n* = 2)	
	5. Helpful for the therapist (*n* = 2) “to help the client”	

Eight patients reported on the unhelpfulness of using the measures. Three patients reported they had found the measures uncomfortable or upsetting, for example, because filling the measures had “opened things up.” Three felt that the measures provided an inaccurate representation of their experience. One patient had used the measures to mislead their psychotherapist. Another patient felt the measures had only been useful for the benefit of their psychotherapist.

These findings provided new data to contribute to the understanding and definition of *patient-perceived helpfulness of measures*, which was used to inform item development as described in Study 2.

## Study 2: Item Development and Initial Psychometric Shortening of the ppHMS

Study 2 consists in the development of items and initial psychometric exploration of the ppHMS.

### Procedure

#### Item Development

We followed methods of inductive scale development to develop items for the construct of patient-perceived helpfulness of measures (DeVellis & Thorpe, 2021). Data themes from patient interviews in Study 1 were used to inform item generation by the first and second author who had immersed themselves in the data. For instance, the first item generated, “I found this form helpful in therapy,” was informed by the first sub-theme in Study 1 “helpful (effective, liked it).” The item “I learnt something from using the form in therapy” was informed by the second sub-theme “supports self-reflection.” Similarly, negatively worded items were derived from unhelpful categories. For instance, our item “This form made me feel down” was based on the Study 1 sub-theme “uncomfortable/upsetting.” The authors also drew on their professional experience and the wider literature to create items: Four colleagues, experts in scale development, were involved in discussions and brainstormed on the definition of patient-perceived helpfulness of measures. The first author conducted an initial review of the literature which was edited by the second author. The first and second author developed additional items based on their knowledge of the literature, experiences as clinicians, and discussions with expert colleagues. This resulted in an item pool of 28 items.

This item pool was refined through a process of expert rating (Hinkin, 2005). A questionnaire with the 28 items was created using the online data collection platform Qualtrics. Four expert psychologists from different theoretical orientations (psychoanalytic psychotherapy, existential psychotherapy, pluralistic psychotherapy, and person-centered psychotherapy)—with published scale development and validation studies, and with experience of form use in psychotherapy—rated each item on DeVellis and Thorpe’s (2021) three criteria: (a) How well it matches the target definition of patient-perceived helpfulness of measures? (b) How well formulated it is for participants to fill in? and (c) How well, overall, it is suited to the measure? They used a 4-point Likert-type scale: 1 = *not at all*, 2 = *a little*, 3 = *moderately*, and 4 = *very well*. An average score was calculated for each item, and those under a cutoff point of 3.0 (items that were suited to the scale less than moderately well) were excluded from further analysis. This resulted in 10 items remaining in the scale.

#### Scale Reduction and Initial Psychometric Exploration

Inclusion criteria to participate in the study were being aged 18 years old and over, currently being in therapy or having attended therapy in the past, and having used questionnaires as part of psychotherapy. Exclusion criteria were being under 18, no experience of psychotherapy, or not having used questionnaires as part of psychotherapy treatment.

Data from three clinical sites and an online convenience sample were collected to drive shortening of the measure. The three clinical sites offered counseling in different psychotherapeutic approaches and of various lengths (6–24 sessions). The clinical sites included the free university psychotherapy research clinic described in Study 1. There were 40 potential psychotherapy patients who were assessed at the free university psychotherapy research clinic, of which 13 did not meet the inclusion criteria or dropped out of therapy before filling out the 10-item ppHMS. The 27 participants at this site completed the ppHMS at Sessions 4, 10, and 24 to rate the PHQ-9, which they had filled out at assessment and then at every session using the data collection software Pragmatic Tracker.

Three participants were recruited from an independent free charity clinic in London and 13 from a commercial clinic in West Sussex, United Kingdom. Participants at these two clinical sites completed the CORE-OM described above (Evans et al., 2002) at every session and the Authenticity Scale, a process measure to assess client authenticity (AS; Lopez & Rice, 2006) at Sessions 4 and 10. The 16 clinical participants filled out the ppHMS at Sessions 4 and 10 either on paper measures or via the online data collection software Pragmatic Tracker. Questionnaires used at clinical sites included basic demographics, the ppHMS for at least one measure used in patients’ current course of psychotherapy, and a single item of form helpfulness.

Finally, 72 participants accessed the survey online via social media platforms (Facebook and Twitter). Of these, 39 exited the online survey without completing the ppHMS. Online participants accessed the Qualtrics survey link posted on U.K. psychology and counseling community pages. The survey was reposted on social media sites over the course of 4 months. The 33 participants in the online sample selected a measure they had used in their psychotherapy: They could choose to rate the PHQ-9, GAD-7, CORE-OM, CORE-10, described above, or the Working Alliance Inventory (WAI), a process measure to assess the bond, agreement on goals and tasks in the psychotherapy (WAI; Bordin, 1979).

In total, 76 participants completed the 10-item ppHMS for at least one measure and were included in the analyses. When participants had completed more than one measure, we included the rating of the first measure they had completed. When participants had completed the measure at several time points, we included the earliest time point (Session 4). The latter decision was to increase the number of possible participants in the study while keeping consistent procedures. Worthington and Whittaker (2006) suggested that *n* should be at least 50 when seeking a single factor. While this might be considered quite small when conducting structural equation modeling, there is strong evidence that for a simple CFA analysis it is sufficient: In an investigation into sample sizes, Wolf and colleagues (2013) conducted Monte Carlo experiments to determine necessary sample sizes for different CFA analyses. They found that, to satisfy a number of criteria (including, for example, a power value of 0.8 or higher), a sample size of 50 was sufficient for a single factor CFA with eight indicators and an average loading of 0.65. In this study, there were 10 indicators and the average loading was 0.70. These figures are very close to the Wolf et al. (2013) numbers, and in both cases, the direction of the small differences would generally lead to a *reduction* in the required sample size. Consequently, we can have confidence that the achieved sample of 76 has a power in excess of 0.8 which is sufficient for the analysis.

### Materials

The survey included the following measures, in this order.

*Patient-Perceived Helpfulness of Measures Scale (10 items)*. The 10 items for the initial iteration of the ppHMS were developed to assess the helpfulness to patients of using measures in the context of their psychotherapy. Items are rated on a 5-point Likert-type scale: 0 = “strongly disagree,” 1 = “disagree,” 2 = “neither agree or disagree,” 3 = “agree,” 4 = “strongly agree.” Example items were “I learnt something from using this form in therapy” and “This form made the therapy better.”

*Single Item of Form Helpfulness*. This single-survey item was used as an acceptability assessment in Cooper et al. (2015). Instructions are as follows: “Please rate the helpfulness of this therapy measure.” The therapy measure is then rated on a 5-point Likert-type scale: 1 = “very unhelpful”; 2 = “unhelpful”; 3 = “neither helpful or unhelpful”; 4 = “helpful”; 5 = “very helpful.” This single item also had the response option of X = “I don’t know.”

*Delighted-Terrible Scale*. The D-T scale is an empirically developed measure of perceived quality of life. It was later validated as a measure of product and service satisfaction (Westbrook, 1980). It is a single-item scale rated on a 7-point Likert-type scale from “delighted” to “terrible”: 7 = “Delighted,” 6 = “Pleased,” 5 = “Mostly satisfied,” 4 = “Mixed,” 3 = “Mostly dissatisfied,” 2 = “Unhappy,” and 1 = “Terrible.” The scale’s instruction is as follows: “How do you feel about ________?.” Instructions in this study were adapted for participants to rate the same measure as for the ppHMS and the single item of form helpfulness. Test–retest reliability ranged from .65 to .84 depending on the service or product being assessed (Westbrook, 1980). The scale had good convergent validity as indicated by strong significant correlations with alternative satisfaction measures within product categories (Westbrook, 1980). Discriminant validity was obtained with small correlations between the D-T scale’s assessment of different products (Westbrook, 1980).

### Participants

Of the 76 participants, 57 (75%) were female, 17 (22.5%) were male, and two (2.5%) chose not to disclose their gender. Ages ranged from 18 to 91 years, with a mean age of 39 years. Participants were predominantly White British (*n* = 55, 72.4%). There were seven participants who identified as White Other (9.2%), five Black British (6.6%), three Asian British (3.9%), two as White Irish (2.6%), and two any other ethnicity (2.6%).

Of the 76, 43 participants used the ppHMS-10 to rate the PHQ-9 (56.5%), 14 rated the AS (18.5%), 12 rated the CORE-OM (15.5%), six rated the GAD-7 (8%), and one rated the WAI (1.5%). When participants completed more than one measure, we only selected a single rating for inclusion in the analysis in order not to repeat cases for the CFA. As the ppHMS is intended to be a generic measure of patient-perceived helpfulness of measures, ppHMS ratings for all measures were combined in the analyses.

### Analysis

#### Scale Shortening Methods and Reliability

Our *a priori* criteria for shortening were as follows: (a) retaining acceptable internal consistency (McDonald’s ω) and (b) reasonable fit to a one-dimensional model, defined as acceptable fit in single-factor CFA without large modification indices for correlated residuals, suggesting a higher order factor structure.

For each of the models, we used the maximum likelihood estimator (MLE) to calculate the parameters and evaluated their adequacy with the chi-square test, the RMSEA, the CFI, and the SRMR. CFI values equal to or higher than .90 and RMSEA and SRMR values lower than .08 indicate an acceptable model fit (DeVellis & Thorpe, 2021).

Shortening was stopped when continuing led to significant deterioration in McDonald’s ω and no improvement on any other parameters in the analyses. The process of shortening, starting with all 10 items, was an evaluation of misfit to the CFA. We removed items which showed most involvement in correlated residuals in the CFA (modification indices for correlated residuals over 10). All analyses were conducted using SPSS v26 and MPlus 8.7.

#### Construct Validity

Construct validity was explored using Pearson correlations for convergent validity with single item of helpfulness and the D-T scale (*r* >.5).

### Results

#### Scale Shortening

**Figure 1.**
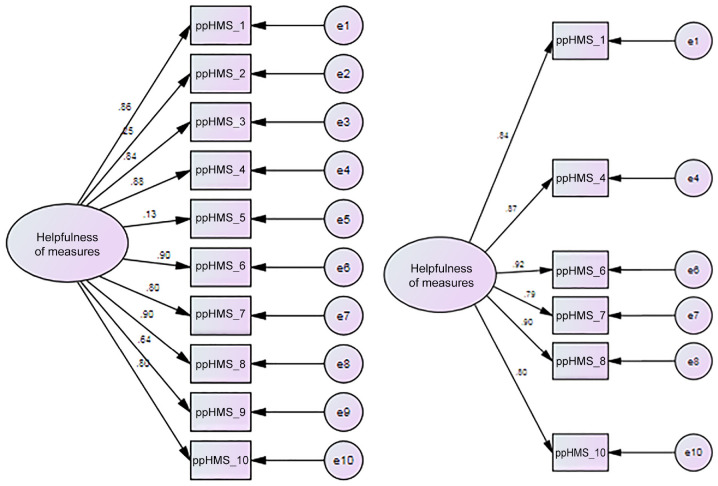
10- and 6-Item Patient-Perceived Helpfulness of Measures Scale (ppHMS) Models.

For all samples combined (*n* = 76), the internal consistency (McDonald’s ω) of the ppHMS-10 was .92. However, CFA indicated that a 10-item univariate model did not have a good fit to the data: χ^2^ (35) = 92.45, *p* < .0005; CFI = .89; RMSEA = .15 (.11, .18) *p*close <.0005, SRMR = .075. We examined factor loadings, correlated items, and the conceptual clarity of items (Figure 1). The two reverse-scored items had very poor standardized factor loadings (Item 2 = .25 and Item 5 = .13), indicating that these items do not fit in a unidimensional model. Examination of the modification indices revealed two further problematic items (Item 9: “Using this form made me think about myself in ways I wouldn’t have done before,” and Item 3: “I would choose to use this form in therapy again.”) Item 9 was highly correlated with Item 10: “I learnt something from using the form in therapy.” We removed Item 9 rather than Item 10 because, as well as having a much lower factor loading (.64), it was the more ambiguous of the two with respect to conceptual clarity. Item 3 (“I would choose to use this form in therapy again”) showed high covariance with Item 1 (“I got something out of using this form”). Again, worse conceptual fit to the construct dictated to remove Item 3. Removal of these four items resulted in a six-item scale with an adequate fit with χ^2^ (9) = 16.83, *p* = .05; CFI = .98; RMSEA = .11 (.001, .18); *p*Close = .11, SRMR = .02. Chi-square was non-significant, CFI above .95, and RMSEA was a little higher than expected; however, the 90% confidence intervals showed promise for this model because the lower bound was .000, but *p* of Close Fit was non-significant, indicating there was a close fit (Kenny, 2015, http://davidakenny.net/cm/fit.htm). Work by Dexin Shi and colleagues (Shi et al., 2019) suggests that when comparing goodness-of-fit measures under different specifications, both small numbers of observed variables (in this Case 6) and small sample sizes (in this Case 76) produce worse goodness-of-fit measures than would be achieved by the same analysis for the total population. Consequently, the figures reported here can be considered as a “worst case scenario” for the underlying fit of the model at a population level.

Internal consistency for the six-item ppHMS was improved with a McDonald’s ω of .95. The impact of the stepwise removal of items from the original 10 items to six items is summarized in Table 2.

**Table 2:** Summary of Fit Statistics From Successive Removal of Factor Items (Study 2)

No. of items	Item(s) removed	ω	RMSEA	CFI	TLI	χ^2^	*df*	Δχ^2^	df (Δ)	*p* (Δ)
10		.919	.150	.898	.864	92.452	34			
8	2, 5	.961	.164	.929	.895	58.015	19	34.437	15	.003
7	9	.951	.138	.962	.938	31.823	13	26.192	6	.001
6	3	.947	.107	.980	.967	16.839	8	14.984	5	.010

*Note.* RMSEA = root mean square error of approximation; CFI = comparative fit index; TLI = Tucker–Lewis index.

#### Convergent Validity

The six-item ppHMS showed a strong significant and positive correlation with the single item of form helpfulness, *r* = .70, *p* < .001 (*n* = 49 [online and clinical samples]).

The six-item ppHMS showed a strong significant and positive correlation with the Delighted-Terrible scale; data were collected in the online sample only and showed high convergence with the ppHMS: *r* = .82, *p* < .001 (*n* = 33 [online sample]).

## Study 3: Replication and Validation in U.K. Stratified Samples

In this study, we used a large U.K. stratified online sample to check that the psychometric parameters from the shortening analyses replicated. Convergence with single-survey items was re-assessed in the new sample. In addition, we assessed measurement invariance across measures, convergent validity with a measure of psychotherapy satisfaction, and divergent validity with a measure of social desirability (Di Malta, et al., 2023). Reliability was assessed using both internal consistency and test–retest reliability with a sub-sample of participants after 1 month (DeVellis & Thorpe, 2021).

### Procedure

Participants who responded to the advert on Prolific.co—an online participant recruitment site—were redirected to the Qualtrics survey platform to complete the study. Participants were invited to provide informed consent prior to completing the six-item ppHMS, STTS-R, Brief Social Desirability Scale (BSDS), and answering a series of demographic questions. Participants were paid the U.K. minimum wage equivalent of 6.5 minutes pro-rata (£0.95) for completing the study, with re-tested participants paid for a further 2 minutes pro-rata (£0.30) upon completing the ppHMS again after a period of 1 month. In this study, the participants used the six-item ppHMS to rate the PHQ-9, GAD-7, CORE-10, and CORE-OM. Three attention check questions were embedded in the questionnaire to avoid low-quality data from bots and “bad faith” participants recruited through Prolific. Data from participants who failed one or more attention check questions were not included in the analysis.

### Material

In this survey, we included the six-item ppHMS, the D-T scale, and the single item of form helpfulness described in Study 2. In addition, we included further socio-demographics questions, a measure of psychotherapy satisfaction, and a social desirability scale.

#### Brief Social Desirability Scale

The BSDS is a brief four-item scale of social desirability (Haghighat, 2007). It is widely used across psychology and other disciplines. It is valid and reliable (Cronbach’s alpha = .60; Turner et al., 2018). Items are answered with “yes” scored 1 or “no” scored 0. Item 4 is reversed scored.

#### Satisfaction With Therapy and Therapist

The STTS-R (Oei & Green, 2008) is a two-dimensional 12-item scale that assesses patients’ levels of satisfaction with psychotherapy. We used this in Study 3 to test convergent validity with patient-perceived helpfulness of measures. The two dimensions include Satisfaction with Therapy (SWT; six items; α = .91) and Satisfaction with Therapist (SWTt; six items; α = .96). The STTS-R has good psychometric properties and a stable factor structure (Oei & Green, 2008). Example items are “I am now able to deal more effectively with my problems” and “The therapist seemed to understand what I was thinking and feeling.” Items are rated on a 5-point Likert-type scale ranging from 1 = *Strongly agree* to 5 = *Strongly disagree*.

### Participants

Participant recruitment was conducted with the aim of achieving a sample representative of U.K. population across categories of age (3), sex (2), and ethnicity (5) in accordance with national census data available at the time (UK-ONS.gov, 2011). The study was advertised to Prolific.co users who were least 18 years of age, resident in the United Kingdom, and answering “Yes” to the Prolific.co screening item “Do you have—or have you had—a diagnosed, on-going mental health/illness/condition?” This latter criterion was a pre-screening process employed to increase the chances that potential participants would meet the criteria of having experience of psychotherapy. Pre-screened individuals viewed an advert that invited participants with experience of being in psychotherapy and had used questionnaires or measures as part of their psychological treatment. Screening questions regarding experience of psychotherapy and form use were part of the survey. Participants who answered “no” to either screening questions were redirected to an end of survey message and excluded from all analyses. The full sample was *n* = 514. Participants’ demographics are detailed in Table 3.

**Table 3:** U.K. Participants Demographics (Study 3)

Participants making non-binary gender selections detailed beside ethnicity	*N* = 514 Average age = 43.39 (*SD* = 14.43)	
	Female *n* = 268	Male *n* = 243
White (2)	234	210
Black (0)	8	9
Asian (0)	18	18
Mixed (1)	5	3
Other (0)	3	3

### Analysis

The ppHMS was designed to work across all measures. The first analysis therefore was to check the invariance of the latent variable structure of the six-item ppHMS across the different measures (e.g., PHQ-9, GAD-7, CORE-10, and CORE-OM). This was achieved by testing both metric equivalence (equal factor loadings across groups) and scalar equivalence (equal intercepts across groups) versus the unconstrained configural model across the groups. CORE-10 and “Other” were combined as they were each too small to include on their own. Analysis was conducted using MPlus v8.6 (Muthén & Muthén, 1998–2017).

### Results

#### Confirmatory Factor Analysis

The results for the five-item CFA indicated model invariance across both measures (Core-10 and Other) for both factor loadings and factor intercepts (Table 4).

**Table 4:** Test for Invariance of Five-Item Latent Variable Across Different Measures (Study 3)

Model	Parameters	χ^2^	*df*	*p*
Configural	60	42.714	20	.002
Metric	48	50.447	32	.020
Scalar	36	71.11	44	.006
Metric vs. configural		7.734	12	.806
Scalar vs. configural		28.396	24	.244
Scalar vs. metric		20.662	12	.056

Having established invariance across measures, it was then necessary to ensure that the total sample model had a satisfactory goodness of fit. The next analysis therefore examined the CFA for the total sample using five of the six items derived from Study 2, excluding variable 2. This gave the following fit indices: χ^2^ (9) = 110.513; *p* < .0005, CFI = .954; TLI = .923; RMSEA =.148 (.124, .173); SRMR = .030. Chi-square, TLI, and RMSEA indices suggested that the model was not optimal; however, CFI and SRMR indices met the required criterion. We examined covariances and found that Item 2 covaried with Items 1 and 3, respectively. Although all items correlated highly with each other, Items 1 and 2 correlated at *r* = .79 and Item 2 and 3 at *r* = .77. All other inter-item correlations were below .7. Inspection of the modification indices in the CFA further indicated high covariance between the error terms of Items 1 and 2. This suggested that Item 2 was potentially redundant in a unique indicator model.

This was further checked by running analyses looking at the impact on the model of removing each item individually (Table 5). This analysis clearly show the significantly greater benefit of removing Item 2 compared with removing any of the other items. The model fit was largely improved with the following fit indices: χ^2^(5) = 22.74, *p* < .0005, CFI = .99; TLI = .98; RMSEA = .083; SRMR = .016. This five-item scale had a much better fit and was more parsimonious.

**Table 5:** Model Fit Statistics Removing Each Item Individually (Study 3)

Item removed	χ^2^	RMSEA	CFI	TLI	SRMR	ω
1	50.76	.133	.972	.944	.024	.905
2	22.74	.083	.990	.980	.016	.899
3	70.69	.160	.960	.920	.028	.904
4	81.36	.172	.958	.917	.028	.916
5	60.49	.147	.967	.934	.028	.904
6	68.00	.157	.964	.927	.027	.907

*Note.* RMSEA = root mean square error of approximation; CFI = comparative fit index; TLI = Tucker–Lewis index; SRMR = standardized root mean square residual.

#### Reliability

Internal consistency for the five-item ppHMS was excellent with McDonald’s ω = .901.

#### Convergent and Divergent Validity

Convergent validity analyses were as expected with moderate correlations with Satisfaction with Therapy *r* = .5; *p* < .0001 and Satisfaction with the Therapist *r* = .58; *p* < .0001. Higher correlations were found with the single item of form helpfulness *r* = .67; *p* < .0001 and the D-T scale *r* = .70; *p* < .0001. Although significant, a much smaller correlation was found with the social desirability scale: BSDS; *r* = .12; *p* < .007, which suggested acceptable divergent validity.

## Study 4: Confirmation of the Five-Item ppHMS in U.S. Stratified Samples

In Study 4, we collected data with three U.S. stratified samples to confirm the new five-item model structure identified in Study 3. We also replicated reliability and validity analyses from Study 3 for each sample.

### Procedures

Study 4 was conducted in the same manner as Study 3, except participants were paid the U.S. minimum wage equivalent of 6 minutes pro-rata (£0.50) for completing the study, with re-tested participants paid for a further 2 minutes pro-rata (£0.20) upon completing the five-item ppHMS again after a period of 1 month.

### Material

In this study, we used the same material as in Study 3, and the participants used the ppHMS to rate the outcome measure PHQ-9, and two other feedback measures: the ORS and the SRS, which are widely used in the United States.

### Participants

Recruitment was also conducted in the same manner as for Study 3, except with the minimum age adjusted to 20 to ensure fit with the relevant national census data (US-CENSUS.gov, 2010). Participants in this study were U.S. residents. Demographics are presented in Table 6.

**Table 6:** U.S. Participant Demographics (Study 4)

Participants making non-binary gender selections detailed beside ethnicity	*N* = 602 Average age = 38.3 (*SD* = 13.64)	
	Female *n* = 296	Male *n* = 273
White (20)	205	196
Black (2)	37	29
Asian (2)	15	12
Mixed (3)	17	16
Other (4)	22	20

### Analysis

All analyses from Study 3 were replicated.

### Results

#### CFA of the Five-Item Model in U.S. Stratified Samples

The absolute overall scores for the five PP-HMS items varied significantly across the three measures, *F*(2) = 10.991, *p* < .001. As a result, it was not surprising that the test for invariance across groups, while demonstrating metric invariance (i.e., the loadings were invariant), did not show scalar invariance (i.e., the intercepts were not invariant) (Table 7). Because this outcome is driven by score variability *across* the measures, the metric invariance supports the hypothesis that the latent ppHMS variable is generalizable for use with different measures.

**Table 7:** Test for Invariance of Latent Variable Across Different Measures (Study 4)

Model	Parameters	χ^2^	*df*	*p*
Configural	45	30.118	15	.012
Metric	37	41.310	23	.011
Scalar	29	58.692	31	.002
Metric vs. configural		11.192	8	.191
Scalar vs. configural		28.573	16	.027
Scalar vs. metric		17.381	8	.026

**Figure 2.**
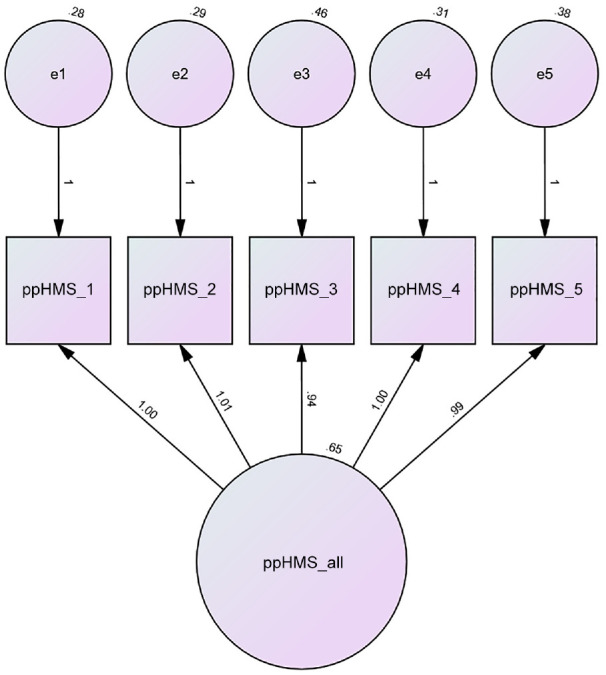
Path Diagram for the Patient-Perceived Helpfulness of Measures Scale (ppHMS) for All Measures Combined *N* = 602.

Fit indices confirmed that the overall five-item model across all measures was a very good fit, χ^2^ (5) = 5.45, *p* ≤ .36, CFI = 1.00; TLI = .90; RMSEA = .01 SRMR = .01. The path diagram for the combined U.S. sample is presented in Figure 2.

#### Reliability, Validity, and Longitudinal Invariance of the Five-Item ppHMS in U.S. Stratified Samples

As with Study 3, the U.S. study involved a second stage of data collection. This allowed us to test for longitudinal invariance, that is, that the ppHMS factor structure remains unchanged over time. As with Study 3, this was achieved by comparing two sets of CFA analysis. In the first analysis, latent variables were created for the two time periods independently. In the second analysis, the loadings and intercepts were constrained to be the same (invariant) across the two time periods. The difference fit statistics between the constrained and unconstrained analyses could then be tested for statistical significance. The results are shown in Table 8 and indicate that there is both metric and scalar longitudinal invariance.

**Table 8:** Test for Longitudinal Invariance of Latent Variable Across All Measures

Model	Parameters	χ^2^	*df*	*p*
Configural	30	25.667	10	.004
Metric	26	33.662	14	.002
Scalar	22	39.388	18	.003
Metric vs. configural		7.995	4	.092
Scalar vs. configural		13.721	8	.089
Scalar vs. metric		5.725	4	.221

Reliability and validity analyses were run for all samples. Analyses suggest that the ppHMS has excellent reliability and validity in the U.S. samples (Table 9).

**Table 9:** Reliability and Construct Validity of the Five-Item ppHMS in U.S. Stratified Samples (Study 4)

	Statistical test	ppHMS_combined *N* = 602	ppHMS_PHQ9 *N* = 200	ppHMS_ORS *N* = 199	ppHMS_SRS *N* = 203
Mean scores		—	*M* = 16.23; *SD* = 4.07	*M* = 17.26; *SD* = 4.18	*M* = 18.16; *SD* = 4.12
Internal consistency	Cronbach’s alpha	.90 [.89, .91]	.89 [.86, .91]	.91 [.88, .93]	.90 [.88, .92]
Test-retest reliability	Pearson correlation	*n* = 306; .56* [.46, .67]	*n* = 102; .52* [.29, .71]	*n* = 102; .56* [.39, .69]	*n* = 101; .60* [.46, .72]
Social desirability (BSDS)	Pearson correlation	.05 [.04, .13]	.09 [.25, .07]	.13 [.02, .24]	.03 [.11, .18]
Satisfaction with therapy (SWT)	Pearson correlation	.43* [.34, .51]	.28* [.12, .43]	.43* [.27, .57]	.56* [.42, .68]
Satisfaction with therapist (SWTt)	Pearson correlation	.48* [.40, .56]	.36* [.21, .49]	.51* [.36, .64]	.56* [.43, .68]
Delighted-Terrible Single item	Pearson correlation	.75* [.71, .79]	.73* [.65, .80]	.73* [.65, .81]	.75* [.67, .82]
Single item of Form Helpfulness	Pearson correlation	.59* [.49, .68]	.50* [.33, .66]	.63* [.46, .78]	.66* [.49, .79]

*Note.* 95% Confidence intervals are given in brackets. ppHMS = Patient-Perceived Helpfulness of Measures Scale; PHQ-9 = Patient Health Questionnaire-9; ORS = Outcome Rating Scale; SRS = Session Rating Scale; BSDS = Brief Social Desirability Scale.

* *p* <.001.

## Discussion

This program of research, comprising four studies, contributes to the development and validation of a brief self-report measure of patient-perceived helpfulness of using measures in psychological treatment. The items for the ppHMS are derived from qualitative interviews with patients and formulated and rated by expert psychologists. The item pool was shortened to a 10-item scale and then to a six-item scale using psychometric shortening in clinical and online samples of psychotherapy patients. Psychometric exploration in a new stratified sample of U.K. patients suggested a five-item model as constituting the best fit. The five-item model was then confirmed in a new U.S. stratified sample. The ppHMS structure was stable across the rating of measures from the most widely used ROM and CL systems in the United Kingdom and the United States: IAPT’s PHQ-9 (Kroenke & Spitzer, 2002), GAD-7 (Spitzer et al., 2006), and CORE-OM (Barkham et al., 2013), and the PCOMS’s ORS (Miller et al., 2003) and SRS (Duncan et al., 2003), suggesting the five-item ppHMS is a generic scale that can be used to rate the helpfulness of psychotherapy measures. The ppHMS had excellent reliability (internal consistency, test–retest reliability), and convergent and divergent validity in U.K. and U.S. stratified samples.

The ppHMS had the highest convergence with the empirically validated D-T scale of product satisfaction (Westbrook, 1980) and medium high convergence with the single-survey item of form helpfulness (Cooper et al., 2015; Fornells-Ambrojo et al., 2017). As expected, convergence with satisfaction with psychotherapy was much lower suggesting some overlap but that the constructs are different (Oei & Green, 2008). Divergent validity with social desirability was excellent with a much smaller correlation (Haghighat, 2007).

The latent construct of patient-perceived helpfulness of measures showed invariance both across different measures assessed and also across time periods. In all cases, both metric invariance (factor loadings being the same) and scalar invariance (mean differences in the latent construct capture all the mean differences in the shared item variance) were observed. This provides confidence that the ppHMS can be used across different measures and also longitudinally over time (Putnick & Bornstein, 2016).

### Limitations

Each study had its own limitations. Study 1 was limited to interviewing participants with no serious mental health conditions or issues of risk, about two symptom measures, a single setting, and to a narrow and homogeneous sample. Relying solely on Study 1’s findings to inform item development may not generalize, miss important constructs, or include superfluous constructs in the item pool, and restrict the scope of the measure. In addition, patients had completed their treatment, which means these patients’ experiences may have been overall positively biased compared to average service users. As a result, these interviews could not provide a representative definition for the construct of patient-perceived helpfulness of measures, only insights on the helpfulness of two widely used measures. We attended to this potential limitation when conducting Study 2 and ensured item development relied on the wider literature and expert input in order for the items to represent a broader understanding of patient-perceived helpfulness of measures. Study 2 was limited primarily by its small sample size, particularly with regard to initial convergent validity analyses but also by the homogeneity of the sample which was predominantly White female. In addition, the online convenience sample constituted the largest proportion of the sample. Overall, this limits conclusions that could be drawn about the sample in this study. Study 3 answered to this limitation with the selection of a stratified sample. This study was limited to rating symptom measures only, and to a U.K. sample, and relied on patients’ recollections of using measures in current or past psychological treatment. The five-item model also needed confirmation in a new sample. Study 4 provided this confirmation but was limited to an online sample of psychotherapy patients reporting on a current or past course of psychotherapy.

Overall, the four studies build on each other to contribute to the validity of the ppHMS. The ppHMS has only been validated in Western samples and would need further validation in diverse groups and in non-western cultures. In addition, as the largest proportion of participants in these studies were responding to an online survey asking them to reflect on their current or past psychotherapy, it is essential that the reliability and validity of the ppHMS are confirmed when using the measure with patients receiving psychotherapy in a clinical setting. The ppHMS was not tested for predictive validity on psychotherapy outcomes, and such study would contribute to extending the validity of the ppHMS.

### Clinical Applications and Future Research

First, the PP-HMS is the only validated measure of patient-perceived helpfulness of measures. As such, at the service level, it can be used to provide patient-centric evidence to inform decision-making around choosing measures as part of ROM and CF systems. It can contribute to existing clinician and researcher criteria for form inclusion (e.g., Lambert & Harmon, 2018).

Second, the ppHMS can be used more specifically as part of research to identify which measures are most helpful and for which patient groups. This may support the implementation of ROM and CF systems which are specifically tailored to specific patient groups. This may be, for instance, based on demographic characteristics or patient diagnoses. Similarly, the ppHMS can be used to compare the helpfulness of measures across different systems or methods of delivery (e.g., weekly, monthly; or online vs. face-to-face) to improve services.

Third, at the individual patient level, the ppHMS can be used in clinical settings to identify whether a patient is finding psychotherapy measures helpful and which are the most helpful measures for the specific patient. According to existing qualitative research on ROM, the themes of flexibility around form use and a preference for measures that capture a patient’s specific issues are recurrent (e.g., Solstad et al., 2019, 2021). Thus, using the ppHMS to select measures that are most helpful can support patient engagement and enhance outcomes through ROM (e.g., Harkin et al., 2016). Future research will need to assess whether integrating evidence as generated by the ppHMS does have an impact on patient outcomes in ROM and CL trials.

The ppHMS can also be used in individual clinical practice to support broader shared decision-making and dialogue between psychotherapist and patient (Faija et al., 2022). Patients have reported that some process measures prompted discussions with their therapist about psychotherapy processes (Cooper et al., 2023; Di Malta et al., 2019). This may be because it gives patients an understanding that such topics are relevant, it gives patients the words and the “permission” to discuss these topics, and as a result it gives them the possibility of finding their voice in these matters. In practice, the ppHMS may prompt dialogue and also point to patient-psychotherapist discrepancies, which would indicate that the psychotherapist may need to spend more time providing psychoeducation about the measure (Faija et al., 2022).

Finally, future research may look at adapting the PP-HMS to be used from the therapist’s perspective (tpHMS). Developing a tpHMS that can be used by therapists would provide additional data and evidence of which measures to include into ROM and CF systems. It may also contribute to addressing issues of clinician engagement with ROM (Lambert & Harmon, 2018).

**Figure 3.**
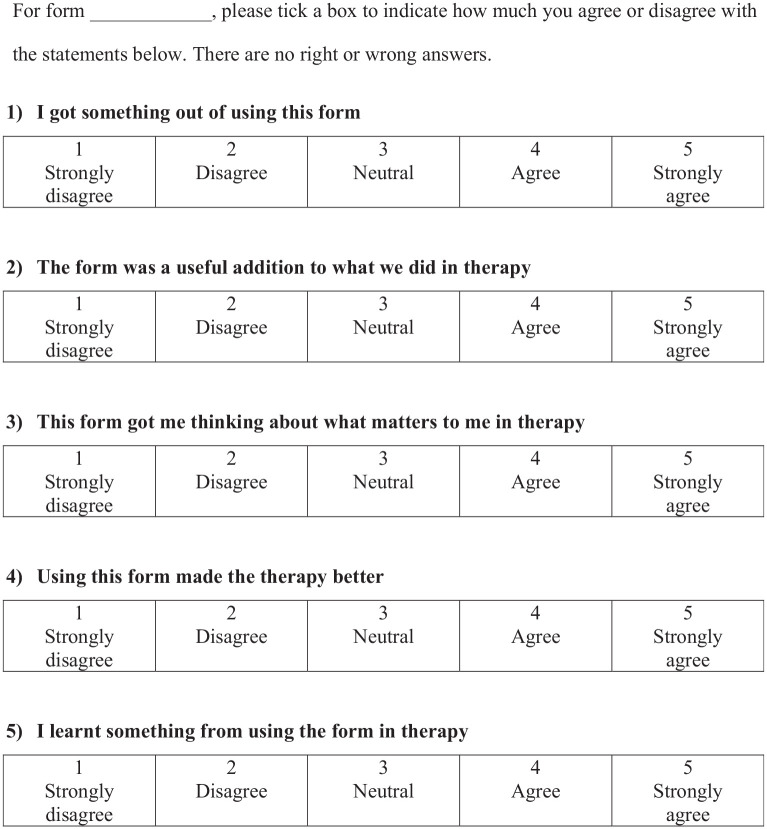
Patient-Perceived Helpfulness of Measures Scale (ppHMS).

The ppHMS (Figure 3) is licensed under the Creative Commons Attribution-NoDerivatives 4.0 International and can be used freely by referring to this research.
